# Diversification or sensory unification? Controversies around the senses in fin de siècle culture

**DOI:** 10.1007/s40656-024-00615-9

**Published:** 2024-03-13

**Authors:** Sonsoles Hernandez Barbosa

**Affiliations:** Universitat de les Illes Balears, Palma, Illes Balears 07122 Spain

**Keywords:** Evolutionism, Sensory discourses, Synaesthesia, Human exceptionality, Oyster, Fin de siècle culture

## Abstract

This article analyses the evolutionist discourses on the senses that emerged in the late 19th century, when theories on the evolution of species were in full sway. Drawing on newspapers, essays and medical literature, this article aims to set face to face the two currents of thought that I have identified regarding sensory evolution: the one that stressed the value of the progressive specialisation of the senses as evidence for human evolution mainly supported by Max Nordau, and the one which regarded the sensory regrouping, exemplified by the phenomenon of synaesthesia, as the true symptom of evolution, strongly supported by Victor Segalen. A close examination of their arguments will provide clues concerning their relative position vis-à-vis the theory that stressed the exceptional nature of humankind among all living beings. Based on newspapers, essays and medical literature, this paper, which straddles several fields (history of science, philosophy, cultural history and aesthetics) aims to set both positions face to face, examining their arguments in detail and establishing their genealogies. This will lead to a better understanding of the scope and range of evolutionist discourses in the fin de siècle culture and on their impact upon artistic practices.

## Introduction

This article will address two different interpretations of the senses in the late 19th century, which were part of the evolutionist discourses that were in full sway at the time, because of the impact of the publication of Charles Darwin’s *On the Origin of Species* in 1859. The article emphasises that discourses about the evolution of species did not refer to living organisms as abstract entities but put the senses at the centre of their arguments. In this context, the body was understood as an entity that engages through the senses – i.e. through its ability to feel pleasure, pain, and even individual freedom – an interface between mind and body, the hinge to interact with the world.

The sensory evolution of species was felt as particularly relevant in a context such as fin de siècle, in which some actors pointed out the problems of the alleged social and cultural progress brought by industrialisation, which wiped out moral, cultural, and aesthetic values rooted in time (Salmi, 2008, pp. 124–125). This conflicted positions about the senses are an illustration of the contradictions that characterised the fin de siècle. The discourses examined here addressed the senses within the framework of debates about the degeneration and regeneration of race, and thus as a reflection of the optimistic and pessimistic views that authors’ held about their own time. The study of the senses will also yield clues about these authors’ relative position concerning the specific exceptionality of humans, a long-held view shared by Platonism, Cartesianism and theology, which evolutionism threatened to undermine. How different positions supported or opposed the historical arguments presented in favour of this thesis will be examined.

The analysis of these discourses about the senses in different European contexts has led me to identify two contradictory positions. On the one hand, authors who stressed the value of the progressive diversification and specialisation of the senses as evidence for human evolution; and, on the other hand, those authors who regarded the sensory regrouping or integration of different senses that synaesthesia implies as the true sign of evolution. Indeed, synaesthesia, a seminal principle of intersensory relations that was beginning to attract the attention of both the medical sciences and artistic practices, played a prominent role in evolutionist discourses. The latter argument was propped up by biological interpretations of synaesthesia, that saw this phenomenon as the zenith of human evolution, and by cultural interpretations which regarded synaesthesia as a deliberately created practice which could be profitably exploited in the creative field. This new paradigm, for which the arts are understood according to their sensorial dimension, and which Sara Danius has identified in relation to Modernist literature (Danius, 2002, p. 1986), links them aesthetically to perception, and therefore to the body in a physiological sense rather than to the intellect, as held by idealist tradition.

The role of the senses in fin de siècle art, and its association with the phenomenon of synaesthesia, have been paid a good deal of scholarly attention (Verna, 2005; Junod, 2006; Hernández Barbosa, 2013; Classen, 2019; Bolpagni, 2021). Here, I shall not focus on the adoption of synaesthesia as an artistic tool, nor on how the arts reacted to debate about artistic practices in such a pivotal moment for them as the early 20th century (Rousseau, 2001), but rather on the way evolutionist discourses affected debates on the role that the senses must play in art. In this way, I am interested in exploring links with natural history theories, and in establishing the genealogy of the different currents of thought. The cross-breeding of thoughts on the senses and evolutionist theories has been paid scarce attention beyond fin de siècle authors, and has only been addressed partially (Forsdick, 2001). I shall thus focus on the way thoughts about the senses bridged theories about human evolution and some aspects of artistic theory related to inter-artistic relations.

The starting point, therefore, are reflections on the senses and their projection onto artistic theory, always in relation to evolutionist theories. The study of the impact of evolutionism, that is, beyond the theory’s strictly scientific merits, in the second half of the 19th century has generated a vast literature; evolutionist theories transcended the scientific field to acquire a social and literary dimension (Halliday, 1971; Belier, 1999; Landry, 2012), especially as a theory of the degeneration of the human species (Chamberlain & Gilman, 1986; Greenslade, 1994). One of the most fruitful avenues of enquiry was that which addressed the study of ‘minor’ species (invertebrates a unicellular organisms), which attracted a good deal of scientific attention in the late 19th century (Schloegel & Schmidgen, 2002; Charpa, 2010). The analysis of this avenue of research has allowed me to establish how molluscs in general and oysters in particular were used as paradigmatic examples of degenerate sensorial perception in evolutionist discourses.

Based on newspapers, essays and medical literature, this paper, which straddles several fields (history of science, philosophy, cultural history and aesthetics) aims to set both positions face to face, examining their arguments in detail and establishing their genealogies. In the next section, I shall address the figure of Max Nordau, the most significant advocate of sensorial diversification. At the time, the oyster was regarded as the least evolved organism, as shown by its lack of physiological diversification, but was nevertheless considered linked to human ontology, as I shall explain in the third section. In the fourth section, I shall examine several theoretical derivations of the notions that regarded sensory regrouping as a symptom of involution, that is, of a regressive evolution towards less developed physiological stages, and the links of this idea with contemporary discourses about the degeneration of race. This will be illustrated by the writings of the Catalonian intellectual Pompeu Gener. In the final section, before the conclusions, I shall present the arguments that held that sensorial regrouping, and its most outstanding expression, synaesthesia, was in fact a symptom of evolution.

## Sensory diversification as a sign of evolution

I shall begin with the current that regarded sensory diversification as the result of the natural evolution of the human species. The notion that organisms evolved towards ever greater diversification had a long tradition. Arthur O. Lovejoy, regarded as the father of the history of ideas, argued that in the late 18th century the system of living beings was conceptualised not as a diversity of static organisms, but as a constant process towards diversification (1983, p. 385). Despite the moralising ideas that often underlie Lovejoy’s arguments (Wilson, 1980; Duffin, 1980), which imply that all species tend to evolve towards perfection, we can agree with him in that Enlightenment thought was already pervaded by incipient forms of evolutionism, for instance in Leibniz’s theories (Lovejoy, 1983, p. 370), which would later be continued by Lamarck, founder of evolutionary theory (Schuller, 2018, p. 8).

Following this line of evolutionist thought, the idea that the evolution of species was directional crystallised in the 19th century, but this was contested by Darwinian theories, insofar as these regard random mutations as a key principle of evolution. The teleological approach to evolution was most clearly laid out in the British naturalist Herbert Spencer’s (1820–1903) works. His *First Principles* (1867) describe evolution as a transit of species from homogeneity to heterogeneity. In Spencer’s perspective, this process was reflected in greater complexity and an increasingly diverse morphology (Frisby, 2014, pp. 79, 150). Applying this to the human species, for Spencer intelligence could be measured by the ability to adapt internal life to external stimuli. This resonated particularly in the 19th century, when living conditions were becoming increasingly heterogeneous and complex (Bonea et al., 2019, p. 13). It was thus understood that evolution moved from the simple to the complex, from homogeneity to heterogeneity. In terms of human evolution, for Spencer this meant overall improvement and better living conditions (Smith, 2019, p. 159). Natural and social and cultural evolution were, therefore, regarded as parallel processes.

This perspective, applied to the relationship between culture and the senses, was adopted by Simon Maximilian Südfeld, publishing behind his pen name Max Nordau. A medical doctor by training, Nordau was one of the most influential intellectual figures in the late 19th century, especially after the publication of his book *Entartung* (Degeneration) in Berlin in 1892. In the following lines I will examine his conceptualisation of the senses in detail.

In *Entartung*, he interpreted many fin de siècle practices –particularly those linked to the symbolist movement– as a source as well as a product of depravation. His criticisms included synaesthesia, on which, as noted above, different sensory fields converged instead of keeping their expressive specificity, in one of the most characteristic features of fin de siècle artistic practice. Applied to literary creation, synaesthesia was mentioned in iconic sonnets such as Baudelaire’s ‘Correspondences’ and Rimbaud’s ‘Vowels’, which drew associations between different sensorial fields. The former recalled ‘perfumes […] sweet as oboes’, linking three sensorial fields (smell, taste, and hearing). However, synaesthesia, as the neurological phenomenon that drives some individuals to spontaneously (i.e. not as a result of learning or creative practice) associate different sensory fields, was not restricted to art, but began in the 19th century to be diagnosed as a subject of medical interest (Hernández Barbosa, 2013, p. 123). It is the neurological condition that Richard Cytowic refers to as ‘perceptual synesthesia’, which is characterised by being ‘automatic, involuntary, and perceptual’ (2018, p. 10). In the fin de siècle, medical synaesthesia attracted so much attention that, by 1895, more than a hundred works had been published on the matter (Jewanski et al., 2020).

Nordau did not agree with this neurological (he referred to it as ‘anatomical’) interpretation of synaesthesia (Nordau, 1892, p. 140). In line with the psychologist Alfred Binet (Binet, 1892, p. 16), he attributed the link between colour and sound to a purely mental association, a product, for instance, of our memories, siding with the psychological interpretation of the phenomenon. According to this position, the phenomenon was available to all artist as a creative tool (Nordau, 1892, pp. 140–141).

Along with this psychological interpretation of synaesthesia, Nordau took a biological approach to the phenomenon, which he regarded as a pathology linked to the idea of degeneration, which was the central concept of his most famous work (Nordau, 1892, p. vii).^1^ The concept of degeneration implies that individuals, institutions and society could not only go forwards, but also go backwards or remain stagnant. In this way, their evolutionary potential was diminished, leading to the cancellation of the species physical and social abilities. This could be due to intrinsic defects, but also by adverse environmental conditions (Hambrook, 2006, p. 1006). Therefore, Nordau’s ideas were a pioneering application of evolucionary ideas to social interpretation presented in Bénédict Morel’s *Traité des dégénérescences* (*A Treatise on Degeneration*, 1857). These notions moved from body-related discourses to social arguments; the concept of degeneration introduced the principle of negative or backwards motion of social evolution.

In Nordau’s case, this degeneration was expressed in certain individuals whose perceptions could be traced back to primitive and unspecialised senses. In this, Nordau sided with the idea that, in the evolution of the species, physiological unity precedes diversification. The return to the form of sensory perception represented by synaesthesia did nothing but reflect ‘this vague intuition of the fundamental unity of essence in all perceptions’ (Nordau, 1892, p. 141). What he is referring to as ‘unity of essence’ is not immediately clear, but in this he seems to be referring to consciousness. Therefore, concerning synaesthetic perception, he argues that ‘consciousness, in its deepest substrata, neglects the differentiation of phenomena by the various senses, passes over this perfection attained very late in organic evolution, and treats impressions only as undifferentiated’ (Nordau, 1892, p. 142); synaesthesia is thus established as a sign of involution. Renouncing the diversifying tool that the senses provide was, for Nordau, the pathological symptom of a degenerate physiology that brings humans back to the sensory conditions of molluscs: ‘it is a descent from the height of human perfection to the low level of the mollusc’ (Nordau, 1892, p. 142).

In consequence, turning synaesthesia into an artistic practice was, for Nordau, a despicable act: ‘to raise the combination, transposition and confusion of the perceptions of sound and sight to the rank of a principle of art, to see futurity in this principle, is to designate as progress the return from the consciousness of man to that of the oyster’ (Nordau, 1892, p. 142). Two ideas stand out in this sentence. First, Nordau points out the degenerate nature of synaesthesia-based artistic creations. This includes the ‘total work of art’ –the proposal of artistic synthesis regarded by the composer Richard Wagner as a revolution– the ultimate expression of this future, degenerate art. Second, the fact that the oyster is attributed consciousness, creating a continuum between the animal and the human being; synaesthesia is a direct nexus with the oyster’s form of primitive consciousness that predates the specialisation of sensory organs.

## The oyster as the least evolved human relative

Nordau insists in this notion of the mollusc as the final stage in the process of human involution. David Hume (1711–1776) had assigned the oyster the role of thinking being with the least developed form of sensory perception, of having, in the words of the philosopher Ricardo Rozzi, the ‘lowest potential for having a mind’ (Rozzi, 2018, p. 194). Indeed, in his *Treatise of Human Nature* (1739), Hume limited the oyster to the ability to feel thirst and hunger. However, in a hierarchy of living beings, despite having such limited sensory capacity, the oyster would share with human beings their consciousness and their ability to adapt in the struggle for survival, the search for pleasure and the avoidance of pain. This led Rozzi to think that Hume places the oyster in the same ontic level as the human being (2018, p. 196). Hume’s conceptualisation of the oyster broke the anthropocentric bias implied in human exceptionality, a principle shared by theological and modern philosophical traditions, which argued for the existence of a categorical gap between humans and other living beings.^2^

As an attentive reader of Hume, Charles Darwin adopted the example posed by the oyster for his theories on the evolution of living beings, in particular concerning the issue of ‘free will’. For Darwin, free will was a common feature of humans and animals, including the oyster. The free will of the oyster rested on those same sensory abilities that Hume attributed to it: ‘Now free will of oyster, one can fancy to be direct effect of organization, by the capacities its senses give it of pain or pleasure.’ (Darwin’s Notebook 1836–1844, quoted in Rozzi, 2018, p. 200). In this way, in the line of thought that goes from Hume to Darwin, the oyster gained a prominent position as an illustration of the living beings’ ability to feel hunger, pain and pleasure, and especially as possessors of free will, and this explain its place as an example of embryonic sensory perception, which also connects this mollusc with the human species. Free will was thus no longer considered an exclusive prerogative of human beings, something reliant on a transcendent psyche or soul that separated the species from the oyster, but a shared trait for species that were part of an ontic continuum.

However, research on the sensory organs of molluscs did not begin until the late 19th century. In 1881, Spengel described the osphradium of bivalve molluscs, the class to which the oyster belongs, for the first time. The osphradium is the sensory organ that molluscs use to detect food; in human terms, this organ comprised the mollusc senses of touch, smell and taste. A few years later, between 1897 and 1901, osphradia were identified also in unibalve molluscs (Haszprunar, 1987, p. 38), such as snails, which illustrates the scientific interest in mollusc sensory systems during this period.^3^

From a different perspective, in the mid-19th century oysters were popular as a Victorian dish. After the publication of Lewis Carroll’s *Through the Looking Glass and what Alice found there* (1871), the sequel of *Alice in Wonderland* (1865), the oyster became, as well as a delicacy, part of the collective imaginary. The novel was a success, and a month after its first publication it had sold 12,000 copies (Carroll, 1995[1871], p. 7). In Chap. 4, the Tweedle twins tell Alice the story of the walrus and the carpenter who, during a walk in the beach, tricked the oysters to follow them, only to eat them afterwards (Carroll, 1995[1871], pp. 82–87). By contravening the laws of normal logic, in Wonderland the differences between humans and animals, including oysters, are erased; animals behave like people and share their tastes, as the walrus demonstrates. The ontic levelling of men and animals –oysters and walruses– is here made explicit. All creatures take anthropomorphic traits, and intermediate categories disappear.

The ontic continuum of beings –the oyster among them– in the Great Chain of Being was already present in *Alice in Wonderland*. In Chap. 3, the idiom ‘You’re enough to try the patience of an oyster!’ (Carroll, 1999[1865], p. 33) is employed. This could be read as portraying the oyster as an eminently passive creature, and thus endowed with enormous patience. The expression is uttered by a young crab immediately before asking her mother to shut up, in another instance of humanised animals. In the process of interpreting Wonderland’s internal logic lead by Alice and the reader, the human nature of crab and oyster are established, although the latter is defined on the basis of its elemental physiology, which separates it even from crustaceans.

Therefore, in the 19th century the oyster was defined as the animal with the most basic sensory capabilities, but still within the same ontic plane as humans. This explains Nordau’s assimilation of humans and oysters: both were part of the same chain, the only difference being the evolutionary stage in which each of them was. This also suggests that Nordau’s link between the oyster nervous system and synaesthesia was not simply an opinion, but ‘an attempt at a really scientific criticism’ (Nordau, 1892, p. viii). The direct connection between the sensory perception of some humans and that of the oyster also found support in the British naturalist Ray Lankester, a key reference for Nordau from whom he even borrowed the title for his book. Lankester’s *Degeneration. A Chapter in Darwinism* (1880) argued that certain species of crustaceans were degenerated versions of more complex organisms which an inverted natural selection process had led to adapt to simpler life conditions (Lankester, 1880, pp. 30, 57). He stated that ‘degeneration may be defined as a gradual change of the structure in which the organism becomes adapted to less varied and less complex conditions of life’ (Lankester, 1880, p. 32). For Lankester, therefore, evolution also was a matter of morphological specialisation and differentiation; in his opinion, the oyster was not an under-evolved species, but an example of involution (Lankester, 1880, p. 56).

The belief in the possibility of human involution places Lankester and Nordau among the followers of the thesis of the ontic continuum of species; humans could degenerate in the same way as any other species. Similarly, the notion that consciousness was not exclusive to humans was another departure from the thesis of human specific singularity, and a major challenge to two of the main arguments of this thesis: the ontic gap between humans and other living beings, on the one hand, and gnoseocentrism, which places theoretical activity as the essence of humanity, on the other (Schaeffer, 2007).

## Discourses about human involution as background to the degeneration of race

From the point of view of human history, the idea that civilisations also undergo periods of decadence was already on the table. Indeed, art history was born under the premise, enunciated by the father of the discipline, Johann J. Winckelmann, that art went through vital cycles: birth, development, maturity and death. This ‘organic metaphor’ model (Davis, 2005) –which explained the progress of civilisations in biological terms– made it easy to explain the fin de siècle as a decadent period caused by the degeneration of the race. We must not forget that the origins of art history as a discipline, including Winckelmann and most 19th century German-speaking historians, was closely related to discourses constructed around the idea of race (Michaud, 2017).

This background, in addition to Morel’s and Lankester’s arguments, made sense of notions that regarded evolution as a process of racial degeneration. This is what Lankester was driving at when he stated that ‘it is well to remember that we are subject to the general laws of evolution, and are as likely to degenerate as to progress’ (Lankester, 1880, p. 60). As early as the 18th century, taxonomies of living beings in which humans were included established race-based hierarchies, which only emphasised a component of animalisation that was intrinsic to racism (Segarra, 2022, p. 29). Moreover, in the fin de siècle the notion of disability associated with sensory experience became basic to argue for racial differences that called for measures of social exclusion. This has led Erica Fretwell to claim that in this context ‘racial difference became a sensory experience’ (Fretwell, 2020, p. 31).

Within this framework, the phenomenon of synaesthesia was seen as a symptom of involution, leading not to specialisation but towards the merge of sensory abilities. For both Nordau and Lankester, the evolution of human physiology had theretofore advanced towards the diversification and specialisation of the senses, so any step in the opposite direction could only be viewed as degenerate.

Max Nordau’s ideas circulated widely in Europe (Hambrook, 2006, pp. 1005–1024). So much so, that his book became ‘the most controversial international best-seller of the 1890^s^’ (Maik, 1989, p. 607). The book was heavily criticised, because of both its cultural and artistic and its biological implications (Cox et al., 1895). Reviews were especially critical of *Entartung*’s tendency to push scientific paradigms too far; the contemporary press was deeply suspicious of Nordau’s approach to science. The critic writing for the North American journal *The Dial* stated that his observations should be examined ‘by a specialist in brain diseases’, that is, that Nordau’s ideas should be pitched against specialised scientific knowledge (Maik, 1989, p. 614). In the 19th century, boundaries between scientific discourse and other branches of knowledge were not always clearly delineated, and medical and scientific postulates were often mixed with philosophical and, as noted, literary ruminations (Otis, 2002), which is not to say that some discourses did not have more legitimacy than others. In the early 20th century, scientific specialisation set in, and discourses became the exclusive abode of specialists working in scientific laboratories (Bowler, 2006, p. 159). Neither Nordau nor any of the other authors that I have mentioned appear to have exchanged ideas with hard-core scientists, including experimental psychologists, whose discipline was only beginning to establish itself, although Nordau cited Wilhelm Wundt, the father of experimental psychology, in the opening pages of his book.

But not all were negative criticisms for Nordau. Numerous fin de siècle intellectuals assumed positions similar to Nordau’s concerning contemporary culture and society. One example is the leading Catalan modernist Pompeu Gener, who was even accused, rightly, of plagiarising Nordau’s ideas (Gras Valero, 2014, p. 77; Vall Solaz, 2018, p. 188).

In line with Nordau, Gener referred to many of the dominating literary and cultural trends of his time as ‘literary pathologies’: symbolism and decadentism, but also Zola’s naturalism, which he regarded as pseudo-scientific in his *Literaturas malsanas. Estudios de patología literaria contemporánea* (Sick Literatures: Study on Contemporary Literary Pathologies, 1894). Gener, like Nordau, attacked symbolists by claiming that ‘symbolism and delinquent decadentism are not a school, but a disease’ (Gener, 1894, pp. 209–210). In contrast with the ‘superior races’ created by natural selection, which he identifies with the ‘Indogermanic or European’ races’ (Gener, 1894, p. 151), some individuals, including the representatives of decadentism, had fallen into a path of physical degeneration in which ‘their brain not only receives external stimuli, but also those that come from deep down, from the organisation of the various organs, the nerves and nervous nodes, such as the spine, the cerebellum, the sympathetic; if such nodes are faulty all representations are wrong’ (Gener, 1894, p. 241). Following Lankester, he claimed that the degeneration of some beings leads to the loss of physiological and mental abilities, such as sensory perception.

For Gener, synaesthesia was also a symptom of organic degeneration. Gener thought that the literature of his time was sick, partially as a result of its use of synaesthesia. Synaesthesia involves a confusion of perceptions –the opposite to a clear perception–, which he linked to the effect of music. In this way, he accused symbolists of ‘negating the idea and equivocating the purpose of literature with that of music’ (Gener, 1894, p. 210). In sensory terms, this meant that for degenerates ‘the notion of things is vague, non-cognoscible, blurred. They mix one with another. One of the first things that they mix up is artistic technique, because of the transposition of perceptions. Sensations are falsified. Coloured sounds make an appearance. Numbers have shapes and colours have sounds or smell’ (Gener, 1894, pp. 239–240).

Gener believed that synaesthesia implies putting sensory perception ahead of the idea, which is thus made indistinct, and that gnoseocentric paradigms should be prioritised. This lack of concretion of the idea is compounded by the arbitrariness implicit to the experience of the phenomenon, because ‘while Rimbaud claims that the I is red, the O blue, and the U green, others say that I is blue, O red and U yellow’ (Gener, 1894, p. 226). In his view, synaesthesia and drugs had a similar effect. It must be pointed out that around 1890 drug consumption –ether and cocaine– was not a marginal practice (Jay, 2000; Gras Valero,^ 2012^). Cocaine, in various forms, was available in all chemists. Drugs, which had been conceptualised in literature by the Symbolists as providing ‘artificial paradises’, and which for Nordau were also a source of degeneration, encouraged a stifled and deformed perception of reality, something that Gener compared to synaesthesia and its pernicious alteration of sensory perception.

In this way, Gener opposes synaesthesia to the vigour and vitality of Nietzsche’s ‘Übermensch’ (Gras Valero, 2014, pp. 78–80), a concept to which he was persuaded to have arrived before Nietzsche (Triviño Anzola, 2000, p. 87). For Gener, ‘supermen’ were created by behaviour, which then reflected upon the nervous system, sensory skills and the brain of some individuals, and which could be inherited. He claimed that ‘through the centuries, training has transformed a rudimentary sensory system into ganglial nodes, and development by development, to histological singularity, a system that is different from everything else; this has reached its peak in the superior being at the upper branches of the zoological tree: man; and into the powerful thought-generating organ, the human brain’ (Gener, 1894, p. 151). According to this, behaviour would end up altering organic material, alterations that can be inherited. This statement by Gener suggests that the natural evolution of human beings ran from basic sensory systems to the more sophisticated biological diversification of the ‘Übermensch’ that characterised superior races, whose maximum expression was the human brain. This development was fundamentally opposed to synaesthesia, which led towards the merge of sensory fields instead of differentiation. This, in Gener’s perspective, was a sign of the degeneration of race.

## Sensory unification as a sign of progress

However, not everyone saw the fin de siècle as a period of sensory-related decadence. In 1902 Ramón María del Valle-Inclán, the leading representative of Spanish modernism in the field of literature, published the following statement in an article about contemporary artistic creation: ‘the characteristic feature of modern art, especially literature, is a tendency to refine sensations and increase their number and intensity’ (p. 114). His text mentioned synaesthesia in the arts; following his interest in the experience of the senses, he regarded synaesthesia as drawing analogies between different sensory fields and appealed to the plastic dimension of music and the musical character of literature. His ideas were rooted in such major figures of French poetry as Rimbaud and Baudelaire cited above as authors of iconic poems for literature’s synaesthetic aspirations. Valle-Inclán also pointed out that synaesthesia was the result of the ‘progressive evolution of the senses’, for today ‘we perceive colour and sound relations which years ago were not there’ (p. 114).

Valle-Inclán thus suggested that sensory perception had reached previously unknown levels of refinement. This refinement was manifested by a more intense apprehension of sensations and a greater ability to establish relationships between various sensory fields, the very definition of synaesthesia. This artistic shift towards sensory exploration was in line with the evolutionist-dominated contemporary biological discourses. It is possible that Valle was aware of Nordau’s evolutionary theories or discourses, which were so well known and commented on by the intellectuals of the time. As such, Valle-Inclán joined a current of thought that examined sensory perception from a phylogenetic perspective, that is, within the broader framework of human evolution, which, in his case, carried a certain component of belief in biological progress.

According to Valle-Inclán, the refinement achieved by the senses by the fin de siècle was at the foundations of the modernist movement of which he was a major figure. Indeed, fin de siècle art –modernism or symbolism, depending on the country– turned sensory perception into a key feature of identity (Hernández Barbosa, 2013; Danius, 2002). In fact, in Valle-Inclán’s works – from *Flor de santidad* (1904) to *Tirano Banderas* (1926)–, the senses played a primary role (Pereiro Otero, 2008). According to this, for Valle-Inclán fin de siècle had witnessed the zenith of sensory perception for the human species and the arts, following a line of biological argument not dissimilar to Nordau’s and Gener’s, but with a very different conclusion.

Valle-Inclán established a link between this sensory progress and the arts of the period, notably the phenomenon of synaesthesia. The idea of sensory unification – and synaesthesia – was for Valle-Inclán the endpoint of the evolution of the senses. In France, synaesthesia became a particularly prominent trope, because medical discourses on this phenomenon became grafted into contemporary cultural trends, especially the symbolist movement. Within the framework of this current, and through the figure of Victor Segalen, we shall see that the sensory unification implied in synaesthesia was interpreted as a sign of progress, in direct and explicit opposition to Nordau’s arguments.

Segalen’s discourse around synaesthesia plays the role of hinge between the clinical sphere and the philosophical-aesthetic field of symbolism. Segalen, who also studied medicine, was a leading French poet during his lifetime, and was close to symbolist circles, especially authors who mobilised synaesthesia in their creative work, such as Debussy, Huysmans, Mallarmé and Rimbaud, as well as the symbolist poet known as Saint-Pol-Roux.

Segalen was awarded a doctorate in medicine with the dissertation *L’Observation médicale chez les écrivains naturalistes* (Medical Observations in Naturalist Writers, 1902). The issue of synaesthesia was addressed specifically in an article, published by the *Mercure de France* in April 1902, entitled ‘Les Synesthésies et l’école symboliste’ (Synaesthesias and the Symbolist School), which revisited some of the major topics of his thesis (Forsdick, 2001, p. 232). This text, which presents his thoughts about the senses within the framework of evolutionist thought, is of particular interest to us.

In this article, Segalen tried to dismantle some widespread prejudices about synaesthesia. He first clarifies that there were two kinds of synaesthesia. First, neurological synaesthesia –Nordau’s ‘anatomical’ synaesthesia, which Segalen referred to as ‘physiological’ (Segalen, 2021[1902], p. 63) and Cytowic ‘perceptual’ – in which the synaesthetic effect is perceived as real by the individual, for instance when the colour evoked by a given musical tone is in fact seen by the listener. Segalen rejects the opinions of the first doctors to deal with this phenomenon, which believed that this form of neurological synaesthesia had a pathological nature (Jewanski et al., 2011, 2020). It was not until the the eighties of the 19th century that doctor Jules Millet finally challenged the prevailing pejorative correlations of synaesthesia (Millet, 1892). Similarly, Segalen opposes the notion that synaesthesia was exclusive to some individuals, a sort of gift related to artistic genius. In this regard, he stated that ‘sensory correlation can be the preserve of the simple or the mediocre’ and that ‘hashish can also bring about these sensations’ (Segalen, 2021[1902], p. 151).

Second, psychological synaesthesia, which Segalen called ‘thought [synaesthesia]’ (‘pensée’), in which the effect is merely imaginary, the product of an association of ideas (Segalen, 2021[1902], pp. 62–63). Contradicting Nordau and Gener, one of the article’s sections firmly states that ‘synaesthesia is not a symptom of degeneration but progress’ (Segalen, 2021[1902], p. 148), emphasising the expressive potential of synaesthesia as a deliberate artistic-literary resource.

In this way, Segalen was convinced that synaesthesias were ‘a powerful —yet intimate— means of art —a prodigious tool—’ (Segalen, 2021[1902], p. 128). Segalen seems to advocate art that uses the sensorial correspondences of synaesthesia far away from the grandiloquence of Wagner’s ‘total work of art’, which involved using all artistic forms of expression to their full potential. Instead, Segalen espouses an intimate form of synaesthesia, illustrated by the use of metaphors. Metaphors are one of his mental vehicles to bridge the gap between literature and matter, between word and chaotic stimuli. Distancing himself from naturalism, which conceptualised the word as a reproduction of reality, in Segalen the word approaches the body through the senses and metaphors. For Segalen, metaphors, incarnated in the mythical past of language, are an example of synaesthetic synthesis, which integrates words – metaphor is a literary resource–, images –since that resource relates two images, one real and another one imagined– and sounds –as verbalisation–; ‘having unified our feelings we can expand the field’ (Segalen, 2021[1902], p. 157). Metaphor allowed the human being to violate nature (‘violons la nature’) (Segalen, 2021[1902], p. 158), so that the implicit artifice of language multiplies the possibilities in the relationship between something and its visual and sonorous expression.

The symbolist theoretician rebukes Nordau for assimilating the notion of progress with sensory diversification, instead of unification, that is, synaesthesia, which for Segalen is an aesthetic aspiration or ideal: ‘sensory correlations […] seem to us, we reaffirm, normal, and suitable for the eternal Procession of Ideas, this great law which succeeds chaos with Analysis, analysis with Synthesis.’ (Segalen, 2021[1902], p. 152). In this way, Segalen, part of a symbolist philosophical trend grounded in idealism (Bouillier, 1996, p. 16; Frangne, 2005), shares an aspiration towards an elemental synthesis, in Mallarmé’s sense of a thought ideal. For Mallarmé, literary text, as illustrated by the *Livre* which he left unfinished with his death in 1898, was based on signs detached from a precise reference but mutually related in reference to an Idea of world shaped as an autonomous cosmos; an idea which, in the case of symbolism, must be understood, within the framework of philosophical idealism, as an act of thought and language (Frangne, 2005, pp. 239, 309; Hernández Barbosa, 2015, pp. 249, 255). In his text, Segalen cites Hegel (Segalen, 2021[1902], p. 86) and Saint-Pol-Roux (Segalen, 2021[1902], pp. 70, 75, 76), and reflects echoes of Mallarmé’s *Livre* in his poetry collection with cosmological resonances *Stèles* (1912).

Segalen’s postulates, like all symbolist thought, are linked to the notion of discontinuity between humans and other living beings. His text on synaesthesia reveals his agreement with three historical arguments for human exceptionality: first, he assumes gnoseological theses that place consciousness as the axis of human condition, be that in the form of awareness, subjective experience of the qualities of things –in relation to the issue of the qualia–, intentionality or self-awareness (Schaeffer, 2007, p. 349). In turn, this argument stands as the foundation of the ontological dualism of humans, in which the body’s material manifestation is accompanied by consciousness as an specific element. This dualism also supported the ontic gap in the order of living beings between humans and other species.

Segalen understands consciousness and language as specific to the human condition. Concerning language as an exclusive trait of the human species, Segalen adopts the principles of the philologist Ernest Renan, an important author for symbolism and a student of the creation of language, in whose process he identifies a synthetic stage as the final goal. This synthesis is the most advanced and superior stage of language, in which, under the rule of consciousness, dialectal variants do without specific peculiarities (Renan, 1988[1848], pp. 184–185).

Within the framework of the dialectical conflict between unity and diversity, mirrored in the literary realm by the dichotomy between naturalism and symbolism, Segalen borrows the concept of synthesis as superior state from Renan. Against evolutionist theories that predicated diversification as a sign of evolution, for Segalen the zenith of evolution was human consciousness (Fourgeaud-Laville, 2002, pp. 53–54). This is also he shared with Gener, for whom the ‘Übermensch’ also crystallised in a superior mind. There was, according to Segalen, no greater evolutionary trait than the crystallisation of human consciousness, able to order the diversity of sensory stimuli and provide it with a single meaning, which was present in the work of art. It is consciousness –the generator of this synthesis and thought synaesthesia– that made humans the pinnacle of evolution. Both currents, therefore, share a gnoseocentric dimension. For both, it is consciousness that draws the line with the animal universe. However, if for Segalen abstraction, the ability to build realities aside from the world, was the most elevated expression of mental activity, for Nordau and Gener this resided in the ability to produce ideas based on the interpretation of the sensory stimuli generated by a complex reality.

## Conclusions

In Nordau’s phylogenetic understanding of the senses, the fin the siècle was a period of decadence. He explained this chiefly as a result of senses that mixed perceptions from different sensory fields, for instance with synaesthesia; instead of moving towards sensory specialisation, assumed as the natural path of evolution, synthesis only brought humans back to a previous evolutionary stage. Spencer’s biological premises held that species evolved towards diversification, but involution, as argued by Lankaster, was also possible. With Morel’s theories of the degeneration of the race and the organicist interpretations of History in the background, figures like Nordau and the Catalan Pompeu Gener regarded sensory synthesis as a sign of the physiological degeneration of the human species. In particular, Nordau illustrated this biological involution with the embryonic physiology of the oyster. At this time, the oyster became an archetype for the most primitive senses possible in a thinking being. This placed oysters and degenerated human beings in the same tier of the evolutionary hierarchy. This view, which connects fin the siècle discourses about the degeneration of race, merges the racialised and the disabled, but also the synaesthete. All of them are linked with the animal dimension, while a more sophisticated evolution, that of the ‘Übermensch’, corresponds to the development of intellect.

Spanish writer Valle-Inclán adopted a similar biology-centred approach, but his conclusions were the exact opposite, regarding the fin de siècle as a period of splendour for the human senses, as illustrated by the mobilisation of synaesthesia by art. Also Segalen understood synaesthesia as one of the most exquisite creative artifices, following its deliberate use by the artist. For Segalen, synaesthesia was the final stage of artistic evolution. His view of synaesthesia as a symptom of progress was grounded on the expanded expressive possibilities that the phenomenon afforded. Through metaphor, synaesthesia multiplied meanings by appealing to more than one sense simultaneously, and liberated language from the straitjacket of communication as a limited means to convey reality. This must be understood within the idealist-symbolist approach to art and the world, for which artistic production aspired to become an autonomous whole operating under its own rules. Synaesthesia thus embodied the most sophisticated formula to construct alternative realities, which situated the human species at the pinnacle of evolution, a categorical different type of being.

If the fin de siècle witnessed the emergence of postulates that began breaking the ontological barriers between human beings and other animals, other discourses, including both those advocating diversification and those that argued for unification, insisted on the exceptional nature of the human species vis-à-vis other living beings, and synaesthesia was mobilised by both, in different senses. In a period in which species such as molluscs and other biological entities tended to adopt human traits –consciousness, but also sociability–, idealist postulates saw sensoriality through synaesthetic sensory perception and the possibilities that idealism attributed the mind –both of which principles were assumed by symbolism– as guarantors of the exceptionality of the human species against the rising tide of theories, so dear to current philosophical anthropology, that advocated the continuum of animal life.

Against idealist arguments, a different approach to sensoriality was gaining ground. The senses were being introduced as a new perceptual paradigm which vindicates the immanence of the body, a relationship with the world that responds to specific perceptual conditions, mostly shared by the animal world, that impose themselves over the mediation of the intellect. This is illustrated by the evolution of artistic disciplines. In the field of plastic arts, the disciplinary inspiration posed by music introduced the possibility of shedding off the need to reproduce sensory reality and embrace abstraction; in that of poetry, it allowed for a model of verse that integrated sonorous and plastic effects at the expense of syntaxis. This opened the door to breaking boundaries between artistic disciplines, in the same way that those between humans and animals began to vanish, as today’s post humanities advocate the ‘animal turn’. As such, in the fin de siècle, the debate about the evolution of the senses, in all its complexity, contributed to blur boundaries: between science and culture; between artistic disciplines; between living species.

## Footnotes

1 A note on terminology is probably in order here. Rousseau refers to Nordau’s ‘biological’ interpretation ‘embriological’; to the thesis that explains synaesthesia as an association of ideas as ‘psychological’; and to the neurological thesis as ‘anatomical’ (Rousseau, 2001, p. 7). I refer to the former as ‘biological’ thesis because I am interested in the theoretical framework on which the argument is based, rather than the phenomenon itself, while referring to Nourdau’s ‘anatomical’ thesis as ‘neurological’, in allusion to the current explanation of the phenomenon in line with Cytowic. The latter is referred to by Segalen as ‘physiological’ (Segalen, 1902, p. 63).

2 The conception of molluscs as our earliest common ancestor is an erroneous historical assumption. Modern science places the origins of evolutionary sensing earlier, in an undersea flatworm, able to sense the world and propel itself (Peter Godfrey-Smith’s, 2021).

3 In addition to molluscs, late 19th century science also undertook the study of protozoa, within the burgeoning interest in the nature of organic beings and in the relationship between physiology and consciousness. The naturalist Ernst Haeckel, a leading figure in this process, argued that protozoa occupied a liminal area between cells and organisms and made a social reading of the matter, by suggesting that this was akin to the boundaries that separated the individual and society (Scholegel, 2002). Following evolutionary theory, he claimed that all organic matter hosted some form of psychological life (Schloegel, 2002, pp. 618, 622).
