# Correction: Diversification or sensory unification? Controversies around the senses in fin de siècle culture

**DOI:** 10.1007/s40656-024-00654-2

**Published:** 2024-12-16

**Authors:** Sonsoles Hernandez Barbosa

**Affiliations:** Universitat de les Illes Balears, Palma, Illes Balears, 07122 Spain

**Correction: History and Philosophy of the Life Sciences (2024) 46:15**

10.1007/s40656-024-00615-9

The Funding information section was missing from this article and should have read ‘This article has been undertaken within the framework of the research project: “Art in its Reverso Context in the period between the 19th and 20th Centuries” (PID2019-105288GB-I00), funded by Ministerio de Economía y Competitividad de España".’

The original article has been corrected.

